# Tension reduction from inter-membrane counter-deformation accelerates nuclear electroporation

**DOI:** 10.1371/journal.pone.0361048

**Published:** 2026-10-23

**Authors:** Yuepeng Diao, Ping Ye, Baolin Liu

**Affiliations:** School of Health Science and Engineering, University of Shanghai for Science and Technology, Shanghai, China; University of Oxford, UNITED KINGDOM OF GREAT BRITAIN AND NORTHERN IRELAND

## Abstract

Pulsed electric field ablation is an emerging non-thermal modality for tumor treatment, in which electroporation of both the plasma membrane and the nuclear envelope plays a critical role in inducing irreversible cellular damage. To elucidate the different effects of nanosecond pulsed electric fields (nsPEFs) on these membranes, we compared the electroporation responses of the single and double membranes under the same field strength employing molecular dynamics (MD) simulations. Our results demonstrate that the double membrane underwent faster electroporation, attributed to inter-membrane counter-deformation under Coulomb force, which modulated the local electric field direction and reduced membrane tension. Compared to the single membrane, the double membrane may be more susceptible to damage induced by nsPEFs. This finding may provide a molecular-scale theoretical reference for understanding the electroporation mechanism of the nuclear envelope relative to the plasma membrane under nsPEFs.

## 1. Introduction

In addition to preserving genetic material and providing structural and mechanical support in eukaryotic cells, the nuclear envelope functions as a critical cellular hub, playing key roles in apoptosis, cellular senescence, gene expression regulation, and cell migration [1]. The integrity and dysfunction of the nuclear envelope are closely associated with tumor initiation and progression, suggesting that regulating nuclear envelope integrity may represent a novel therapeutic target for cancer treatment [2,3].

In recent years, nsPEFs have emerged as a novel modality for cancer treatment [4–6]. The nanosecond to microsecond timescale of pore formation makes real-time observation challenging. However, advances in multi-scale computer simulations now enable predictive modeling of electroporation across tissue, cellular, and molecular levels. At the tissue scale, finite element simulations using patient-specific glioma models demonstrated that high-frequency irreversible electroporation improved the ablation of gliomas with varying sizes and shapes while reducing the risk of muscle contraction associated with conventional irreversible electroporation [7]. At the cellular scale, automated modeling of realistic, large-scale systems enabled more accurate simulation of the intercellular interaction, improving the prediction of electroporation therapy outcomes [8]. At the molecular scale, MD simulations demonstrated that solvent molecules at the bilayer-water interface reorient rapidly on picosecond timescales under the electric field, promoting pore formation within nanoseconds. Moreover, molecular dynamics and finite element methods showed close agreement in the extent and area percentage of electroporated plasma membrane under the same external field [9]. Guo et al.[10] showed that electric-field-induced membrane porosity lowered the local Young’s modulus. This reduced resistance to electro-deformation. It indicates that membrane structural changes regulate cell deformation via a mechanical feedback loop.

Studies have shown that nsPEFs not only induce electroporation of the plasma membrane but also penetrate the membrane to directly affect intracellular structures, causing electroporation of organelle membranes such as the nuclear envelope [11,12]. Similar to plasma membrane electroporation, nuclear envelope electroporation is the process by which the external electric field increases the permeability of the nuclear envelope, leading to the formation of nanoscale pores [13]. Current studies aimed to understand the parameter sensitivity of plasma membrane and nuclear envelope electroporation under nsPEFs [14–16]. The pore formation induced by electroporation of the nuclear envelope is similar to that of the plasma membrane, but specific studies at the nuclear envelope level are still ongoing [17]. Weaver et al. conducted simulation studies using the mesh transport model and found that nsPEFs can simultaneously affect both the plasma membrane and the nuclear envelope [18]. Compared to electroporation of the plasma membrane, nsPEFs induce greater structural damage to the nucleus, mitochondria, and endoplasmic reticulum [19]. Alterations in osmotic pressure or mechanical compression can drive lipid rearrangement to reduce membrane tension, consequently leading to nuclear envelope rupture and genomic instability, which ultimately impacts cell proliferation, migration, and resistance to anticancer therapies [20–22]. Current electroporation strategies primarily target the plasma membrane. Given that cancer cells often exhibit enlarged nuclei and increased nucleocytoplasmic ratios [22], targeting the nuclear envelope as a novel electroporation site could improve the efficacy of cancer treatments.

To elucidate the different effects of nsPEFs on the nuclear envelope and plasma membrane at the microscopic mechanistic level, this study employed MD simulations to analyze the electroporation effects in the single and double membranes under the same electric field strength. The study focused on the regulatory mechanisms of Coulomb force and membrane tension variations induced by the electric field on the electroporation process. Simulation results demonstrated that, under the same electric field conditions, the double membrane required less time to form pores than the single membrane. This is attributed to the unique counter-deformation in the double membrane driven by Coulomb force, which altered the local electric field angle distribution and consequently caused a significant reduction in the membrane tension, thereby facilitating electroporation. Compared to the single membrane, the double membrane exhibited higher electroporation efficiency under nsPEFs. This finding contributes to understanding the electroporation behavior of the nuclear envelope under nsPEFs.

## 2. Materials and methods

The primary difference between the nuclear envelope and the plasma membrane is that the nuclear envelope consists of the double membrane, whereas the plasma membrane is composed of the single membrane. During the construction of the nuclear envelope model, the CHARMM-GUI was employed to assemble a model consisting of two phospholipid bilayers, which was immersed in a 0.15 M NaCl solution approximating the ionic concentration of extracellular fluid. Each phospholipid bilayer contained 1024 1,2-Dipalmitoyl-sn-glycero-3-phosphocholine (DPPC) molecules, and the intermembrane space of the nuclear envelope was idealized as being filled with 0.15 M NaCl solution. In parallel, the plasma membrane model composed of a single phospholipid bilayer containing 1024 DPPC molecules was constructed and placed in a 0.15 M NaCl solution. Due to the micro- to nanoscale scope and computational constraints of MD simulations, we employed a coarse-grained model with the same DPPC bilayer structure for both membrane systems. The analysis focuses on their most fundamental structural difference—the number of phospholipid bilayers—allowing for the controlled comparison of electroporation effects. Moreover, phosphatidylcholine is the major phospholipid component of mammalian cell membranes [23], and DPPC has been widely used in the MD simulations of the membrane systems [24–26]. The molecular structures of both model systems are shown in Fig 1. The system dimensions were 12.5 × 17.3 × 17.3 nm³ for the single membrane and 50.8 × 17.3 × 17.3 nm³ for the double membrane. Under periodic boundary conditions, the system is equivalent to an infinitely extended membrane [24,27]. The lateral dimensions of the membrane in this study are comparable to those used in previous work [24]. The intermembrane distance between the upper and lower phospholipid bilayers of the double membrane was set to 21.6 nm, consistent with previous research [28].

**Fig 1:**
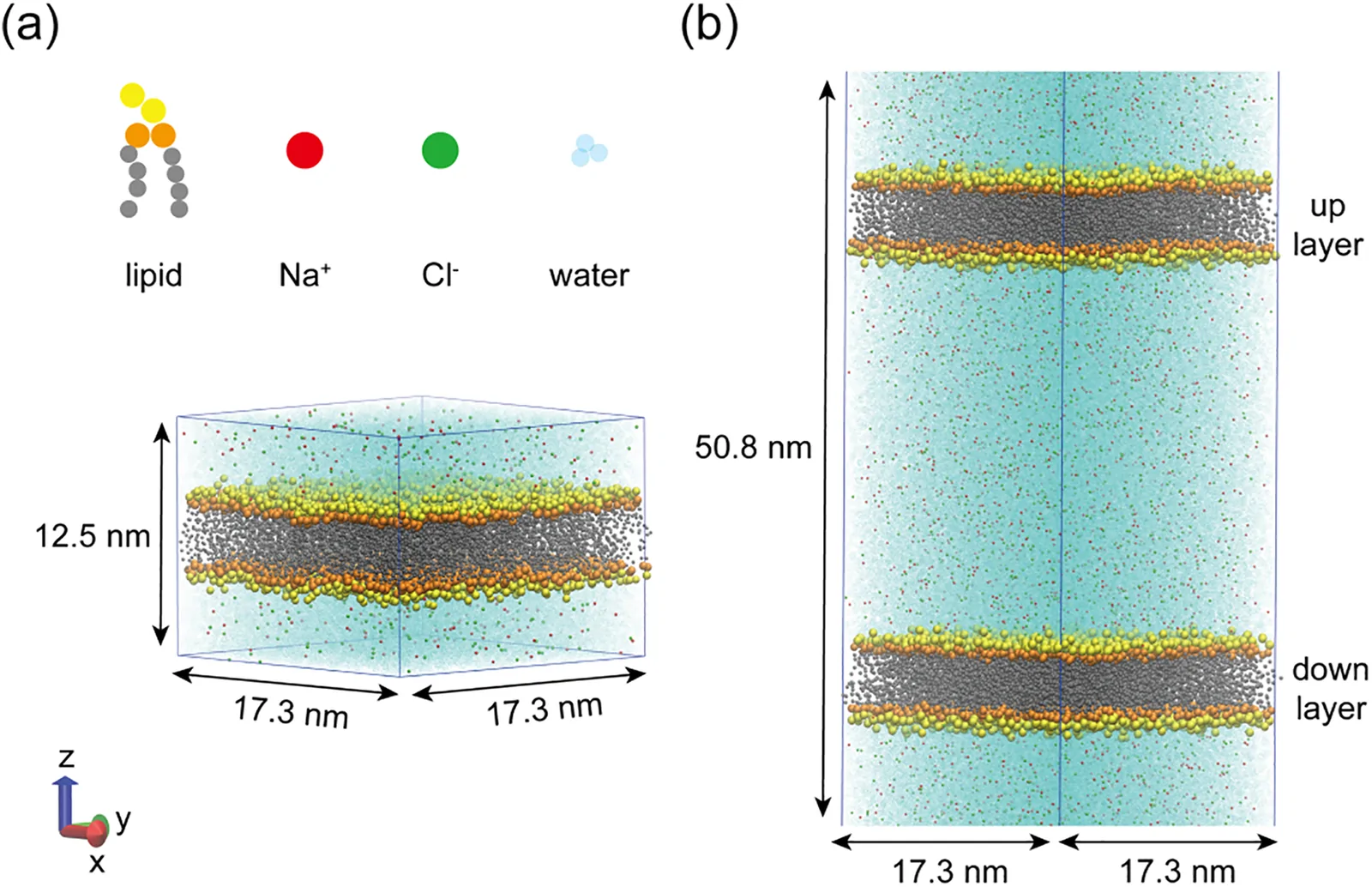
System structure. (a) The single membrane. (b) The double membrane. Orange, yellow, and gray particles represent the phospholipid membranes, while light blue, red, and green particles represent water molecules, Na⁺ ions, and Cl^-^ ions, respectively.

All the simulations were performed using the GROMACS simulation package [29] at the coarse-grained level with the Martini force field [30–32], and periodic boundary conditions was applied in all three dimensions [24]. In the MD simulations, the equations of motion were integrated using the leapfrog algorithm. During the pre-equilibration process, both the single and double membranes first underwent 5000 steps of energy minimization with a step size of 10 fs, followed by 100 ns of NPT equilibration [33]. The temperature was maintained at 310 K using the Berendsen algorithm [34], and the pressure of 1 bar was coupled semi-isotropically using the Parrinello-Rahman algorithm [35]. Finally, the 50 ns NVT equilibration was performed [36], with the temperature maintained at 310 K using the V-rescale algorithm [37]. Subsequently, constant electric fields with strengths of 0.1, 0.2, 0.3, 0.4, 0.5, and 0.6 V/nm were applied to the single and double membranes. The selected electric field strengths fall within previously reported ranges [27,38]. The upper limit of 0.6 V/nm ensures stable pore formation within accessible simulation timescales and prevents rapid membrane collapse under excessively high fields. Under electric fields far exceeding physiological thresholds, the field drives water into the hydrophobic regions of the membrane, where continuous water channels form on picosecond-to-nanosecond timescales, markedly faster than in conventional electroporation [39]. The pore formation time was measured from the application of the electric field. Notably, the external electric field was applied along the membrane normal (i.e., the z-axis perpendicular to the membrane surface) by exerting the additional force on all charged atoms in the simulation system according to the predefined electric field vector [40,41]. Since the analysis of membrane tension response relies on the pressure tensor method [42], the production phases of all simulation systems were performed under the NVT ensemble, consistent with existing studies [43–45]. Additionally, compared to the charge imbalance method, the external electric field method enables precise control over the transmembrane potential to near-physiological ranges [46]. The resulting linear potential profile also exhibits minimal influence from local ion fluctuations, leading to superior reproducibility and broad adoption in simulations of membrane electroporation [15,47,48]. Three independent simulations were performed for each electric field configuration in the single and double membranes. All analyses, except for pore formation time, were performed using trajectory data within a 1 ns window centered around the pore formation event. Each simulation was conducted for a duration of 20–50 ns. Short-range electrostatic and Lennard-Jones interactions were truncated at 1.2 nm, while long-range electrostatic interactions were calculated using the Particle Mesh Ewald method [49]. All simulations were performed with a time step of 20 fs, and coordinates were saved every 0.1 ns. Molecular images were generated using VMD [50]. Given that the free-energy barrier for water entry into the hydrophobic region of the phospholipid bilayer is much lower than that for ions [51], the external electric field preferentially drove water molecules into the hydrophobic region, forming a continuous water wire. Simultaneously, lipid headgroups reorient toward the water wire to form the hydrophilic pore, allowing more permeation of water molecules and ions [52]. Therefore, the time of pore formation was defined by the first observation of a continuous water wire spanning the hydrophobic region of the phospholipid bilayer [53]. Trajectory analysis was conducted using built-in tools from the GROMACS software package. Membrane tension was calculated by extracting the pressure tensor using the “gmx energy” command [42].

## 3. Results and discussion

### 3.1 Facilitated pore formation in the double membrane system

To investigate the different damage induced by nsPEFs in the single and double membranes, we employed MD simulations to compare their electroporation effects by applying constant electric fields to both membrane systems. The results revealed that, under the same electric field strength, the double membrane consistently required less time to form pores than the single membrane (Fig 2). The pore formation times were consistent across the three replicate simulations for both single and double membranes. As the electric field strength increased from 0.2 V/nm to 0.6 V/nm in increments of 0.1 V/nm, the pore formation time of the double membrane decreased from 0.6 ns to 0.2 ns, while that of the single membrane decreased from 4.6 ns to 0.3 ns. At the same electric field strength, pore formation time differed significantly between the two membrane systems (p < 0.0001). The time difference required for the pore formation between the double and the single membranes also gradually decreased with increasing electric field strength, consistent with the previous reports [54]. The results suggest that the nuclear envelope with the double membrane is more susceptible to electroporation than the plasma membrane with the single membrane, which is in accordance with the conclusions of the previous study [19]. Moreover, the difference in electroporation efficiency between the two membrane systems was particularly pronounced at lower electric field strengths. This may be related to the significantly higher electric force acting on the nuclear envelope than on the plasma membrane [19], which may allow the double membrane to more easily overcome the electroporation energy barrier at the lower external field strength, leading to faster pore formation than in the single membrane.

**Fig 2:**
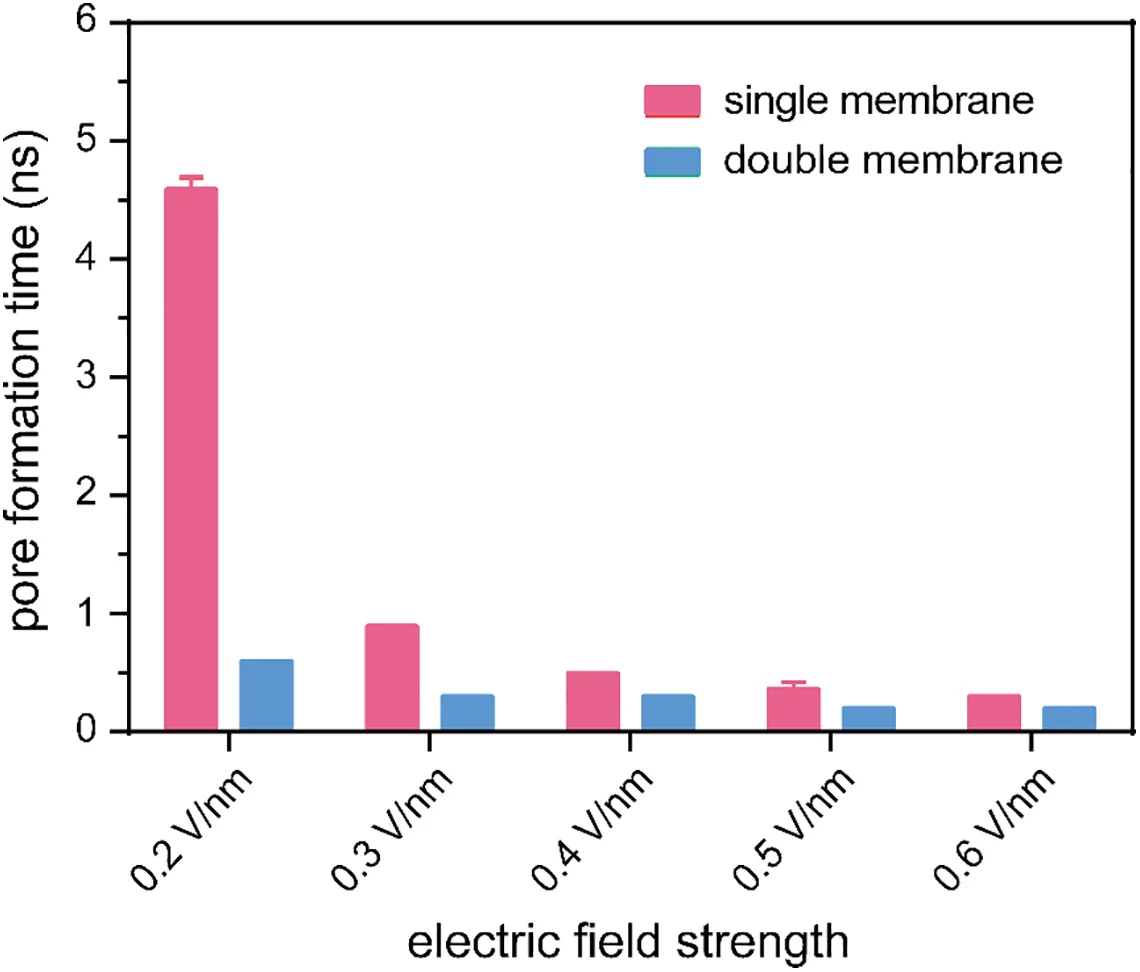
Comparison of the pore formation time between the single and double membranes under different electric field strengths. The pink and blue bars represent the single membrane and double membrane, respectively. Error bars represent standard deviation.

To further assess the potential effects of water-layer thickness under periodic boundary conditions and coarse-grained force-field selection on the simulation results, we constructed a thickened-water membrane system and an all-atom membrane system, respectively, using the single membrane system as the reference model (see the Supporting Information, Section 1.1). Under the same electric field conditions, neither system showed a significant difference in pore formation time compared with the single membrane system, indicating that water-layer thickness and force-field selection do not significantly affect the comparison of pore formation times between the single and double membrane systems (see the Supporting Information, Sections 1.2 and 1.3).

Further pore characterization revealed that, under the same electric field conditions, the double membrane exhibited a greater number and larger size of pores compared to the single membrane (Fig 3), consistent with the previous study [19]. Additionally, both pore number and size increased significantly with the rising electric field strength, aligning with the previous report [55]. Furthermore, in the early stage of pore formation, the pore positions do not necessarily correspond. Over longer timescales, multiple adjacent pores are observed to gradually expand and eventually coalesce, forming interconnected channels. As the pores enlarged, however, those on the upper and lower phospholipid bilayers of the double membrane tended to align, forming continuous channels that enabled bidirectional solution transport. Notably, membrane sensitivity is not determined by lipids alone. Membrane proteins, especially ion channels, act as field-sensitive sites [56] and differ from electroporation in pure lipid systems. Future work should develop more realistic composite cell or nuclear envelope models.

**Fig 3:**
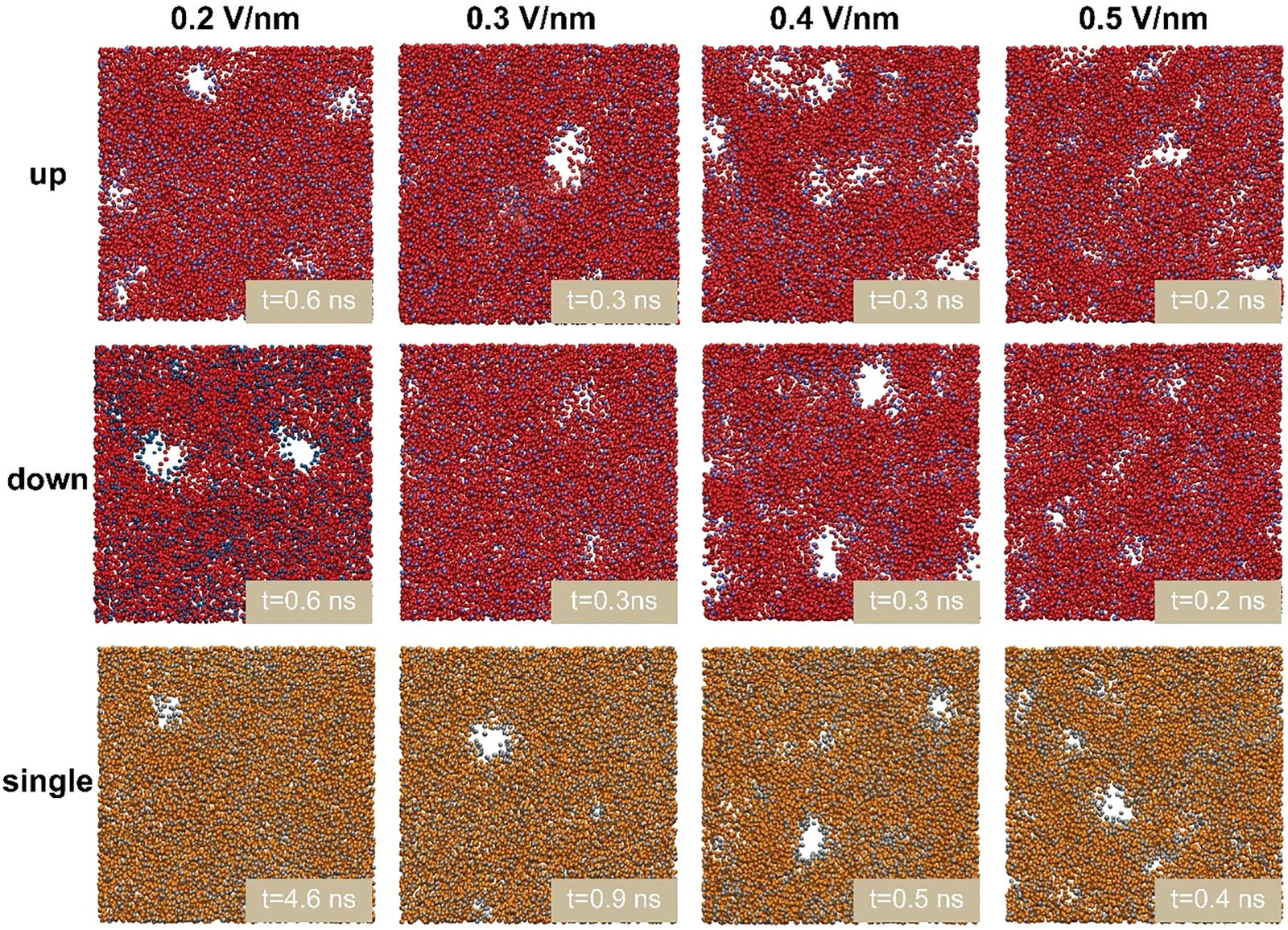
Top-view VMD snapshots of pore formation in the single and double membranes under different electric field strengths. “up” and “down” denote the upper and lower phospholipid bilayers of the double membrane, respectively, while “single” represents the single membrane.

### 3.2 Pore formation facilitated by coulomb interactions modulated by inter-membrane counter-deformation

To compare the electroporation kinetics between single and double membranes, we quantified the pore area ratio within the 0.2–0.6 ns time window under the electric field strengths ranging from 0.2 to 0.6 V/nm. The pore area was quantified as the fraction of grids unoccupied by atoms in the XY-plane projection. Specifically, the XY-plane of the system box was divided into 0.43 nm × 0.43 nm grids, a scale comparable to the characteristic size of hydrophobic pores that accommodate a single water column [51]. The grid ratio without atoms was calculated at various time points (0.2–0.6 ns) and electric field strengths (0.2–0.6 V/nm). A region is determined as “atomic” if the center of mass of any atom belonging to a phospholipid molecule falls within that grid cell. The results showed that under the same conditions, the double membrane consistently exhibited a larger pore area ratio than the single membrane, demonstrating that it is more susceptible to electroporation (Fig 4). At 0.6 ns, the pore area percentages of the single and double membranes were 0% vs 0.53% (0.2 V/nm), 0.06% vs 12.60% (0.3 V/nm), and 0.56% vs 13.53% (0.4 V/nm), respectively. At 0.4 ns, the corresponding values were 0% vs 3.79% (0.3 V/nm), 0% vs 5.28% (0.4 V/nm), and 0.31% vs 8.16% (0.5 V/nm). At 0.2 ns, no pore formation was observed in the single membrane within the electric field strength range of 0.4–0.6 V/nm (all percentages 0%), while the pore area percentage of the double membrane increased from 0.03% (0.4 V/nm) to 0.75% (0.6 V/nm) with increasing electric field strength. For both membrane systems, the pore area increased with prolonged electric field exposure, consistent with the previous report [57].

**Fig 4:**
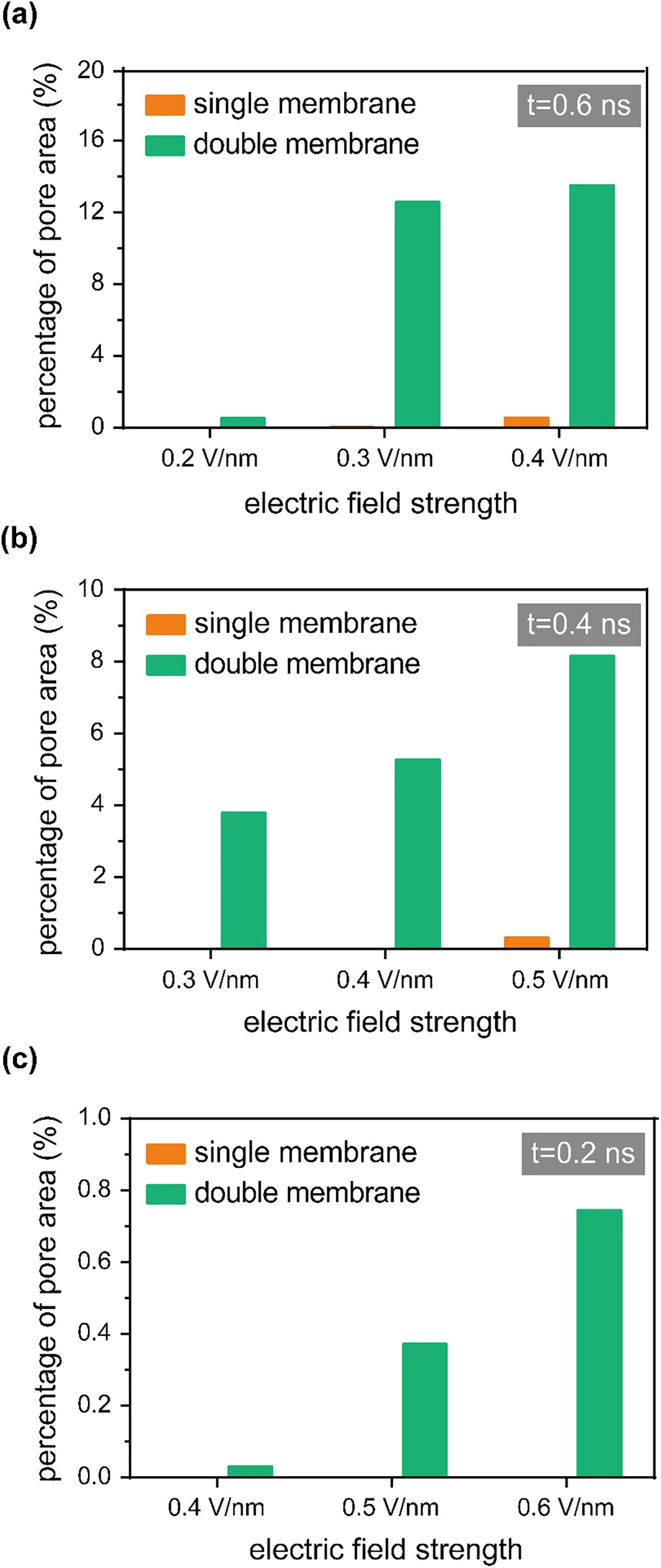
Comparison of pore area ratios between the single and double membranes under different time points (0.2, 0.4, 0.6 ns) and electric field strengths (0.2-0.6 V/nm). (a) 0.6 ns; (b) 0.4 ns; (c) 0.2 ns. The time measured from the application of the electric field. The orange and green bars represent the single membrane and double membrane, respectively.

Given that the Coulomb force is the direct driving force for electric field-induced pore formation [58], we analyzed the Coulomb force experienced by the single and double membranes along the XYZ directions. Specifically, the force along the Z direction was obtained by summing the Z-components of the Coulomb force of individual phospholipid molecules, while that in the XY plane was derived from the vector sum of their X and Y components. These calculations adhere to Coulomb’s law [59]. The results showed that, under the same electric field strengths, the double membrane experienced lower Coulomb force in the XY plane (perpendicular to the membrane normal) compared to the single membrane (Fig 5a), while exhibiting higher Coulomb force along the Z direction (parallel to the membrane normal) (Fig 5b). Specifically, as the electric field increased from 0.2 V/nm to 0.6 V/nm, the Coulomb force in the XY plane decreased from 3.27 × 10^5^ to 2.52 × 10^5^ kJ/mol/nm for the double membrane, and from 3.70 × 10^5^ to 2.97 × 10^5^ kJ/mol/nm for the single membrane. Conversely, Coulomb force along the Z direction increased from 61.48 to 132.74 kJ/mol/nm for the double membrane, and from 56.88 to 124.91 kJ/mol/nm for the single membrane. The results indicate that under the influence of an electric field, the double membrane is more effective than the single membrane in reducing the Coulombic interactions of phospholipid molecules perpendicular to the membrane normal, while enhancing those parallel to the membrane normal, thereby creating conditions favorable for pore formation. Additionally, as the electric field strength increases, both membrane systems exhibited a decrease in Coulomb force perpendicular to the membrane normal and an increase in that parallel to the membrane normal. This suggests that Coulomb force perpendicular to the membrane normal hindered pore formation, while that parallel to the membrane normal facilitated it, consistent with previous research findings [60].

**Fig 5:**
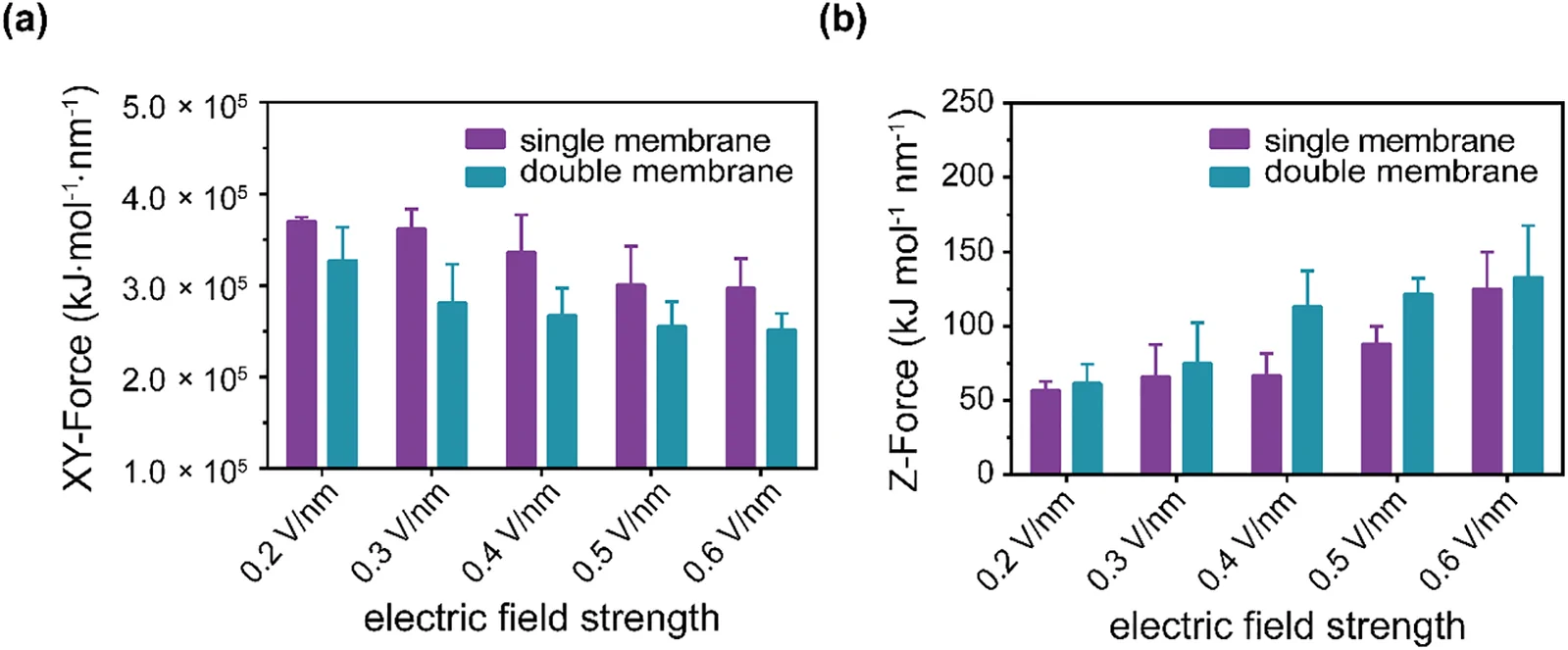
Comparison of Coulomb force experienced by the single and double membranes in the XYZ directions under different electric field strengths. (a) Comparison of Coulomb force in the XY plane perpendicular to the membrane normal for the single and double membranes; (b) Comparison of Coulomb force in the Z direction parallel to the membrane normal for the single and double membranes. The purple and blue bars represent the single membrane and double membrane, respectively. The error bars represent the standard deviation of three independent simulations. For each simulation, values were averaged over a 1 ns time window centered on the pore formation time.

To demonstrate the reliability of the results, we have included in the Supplementary Materials the Coulomb force in the XYZ directions and membrane tension data from the three replicates for both single and double membrane systems under the 0.2 V/nm electric field. In the single membrane, the XY-direction forces were 3.70 × 10^5^ kJ/mol/nm, 3.76 × 10^5^ kJ/mol/nm, and 3.79 × 10^5^ kJ/mol/nm (S4a Fig in S1 File); the Z-direction forces were 56.88 kJ/mol/nm, 55.83 kJ/mol/nm, and 56.48 kJ/mol/nm (S4b Fig in S1 File). In the double membrane, the XY-direction forces were 3.26 × 10^5^ kJ/mol/nm, 3.27 × 10^5^ kJ/mol/nm, and 3.29 × 10^5^ kJ/mol/nm; the Z-direction forces were 61.51 kJ/mol/nm, 62.93 kJ/mol/nm, and 62.20 kJ/mol/nm. These results indicate no significant differences among the three replicates for each system. In addition, we analyzed the lipid headgroup angle, membrane thickness, and water permeability around headgroups under different electric field strengths in the single membrane. The headgroup angle is defined as the angle between the PO4/NC3 vector and the membrane normal (i.e., the electric field direction). Without an applied field, the angle distribution peak was at 90°. When the field increases from 0.2 V/nm to 0.6 V/nm, the peak shifted to smaller angles, decreasing from 75° to 30° (Fig 6a), indicating that lipid headgroups become more aligned with the electric field [47]. This reorientation may contribute to an increased Coulomb force component parallel to the membrane normal between phospholipids and a reduced perpendicular component. The mass density profiles along the membrane normal also indicated no obvious variation in water distribution (Fig 6b). Membrane thickness remained constant at ~4.26 nm, suggesting that the electric field does not significantly affect headgroup hydration, water permeability, or overall membrane thickness. In addition, membrane deformation mechanics have been shown to be strongly dependent on lipid type (tail length, head group, saturation, etc.). In this study, DPPC was used as a model membrane system, which has been widely applied in studies of electroporation and membrane disruption and can effectively capture field-induced pore formation [27,38]. Future work will consider more complex, cell-type–specific membrane models (e.g., cancer cells) incorporating lipid type and composition-dependent concentrations.

**Fig 6:**
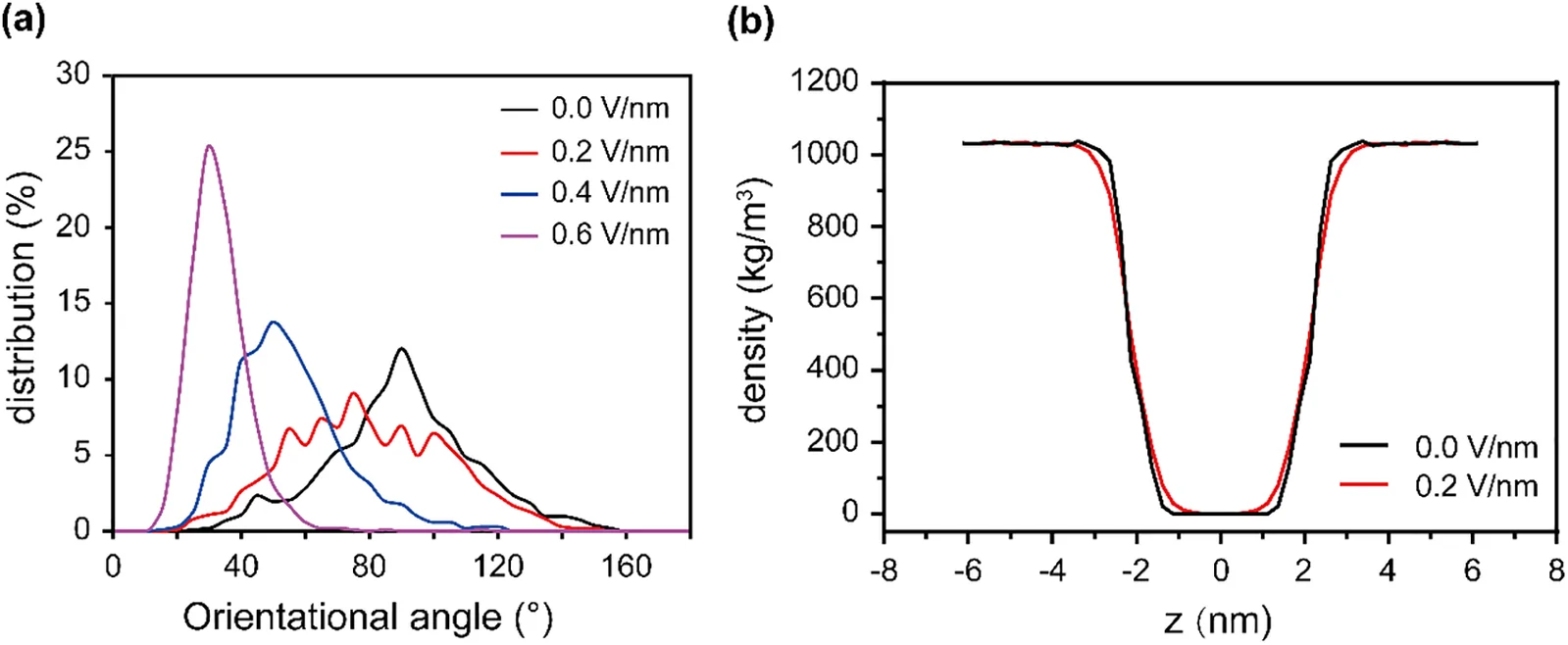
Effect of electric field on phospholipid headgroup orientation and water density distribution. (a) Distribution of lipid headgroup orientation angles. Black, red, blue, and purple curves represent electric field strengths of 0.0, 0.2, 0.4, and 0.6 V/nm, respectively. (b) Water mass density distribution along the membrane normal. Black and red curves represent electric field strengths of 0.0 and 0.2 V/nm, respectively.

The application of an external electric field induces the field-driven force on the membrane surface, leading to membrane deformation and promoting pore formation [61]. To investigate whether the differences in Coulomb force between the double and the single membranes arise from membrane deformation under the electric field, we used VMD to compare their pore formation characteristics (Fig 7). Under periodic boundary conditions, the single membrane exhibited a uniform deformation, whereas the double membrane showed counter-deformation between the upper and lower bilayers. To verify the significance of the deformation in the double membrane compared with the single membrane under the same electric field, the equilibrium position without an electric field was used as the baseline. We analyzed the fluctuation amplitude along the electric field direction for both membrane systems at 0.6 V/nm. The results show that the fluctuation amplitude of the double membrane is approximately 3.1 times that of the single membrane relative to the baseline, indicating that the double membrane exhibits stronger fluctuations than the single membrane. This behavior is not consistent with typical thermal motion of the system. This resulted in local regions where the two bilayers approached each other, with pores predominantly forming near these zones of proximity. In the double membrane, such inter-membrane deformation may locally reduce the angle between the membrane normal and the direction of the electric field, causing the electric field angle distribution to shift toward smaller values. This shift enhances the Coulomb force component parallel to the membrane normal while reducing the perpendicular component, thereby facilitating pore formation.

**Fig 7:**
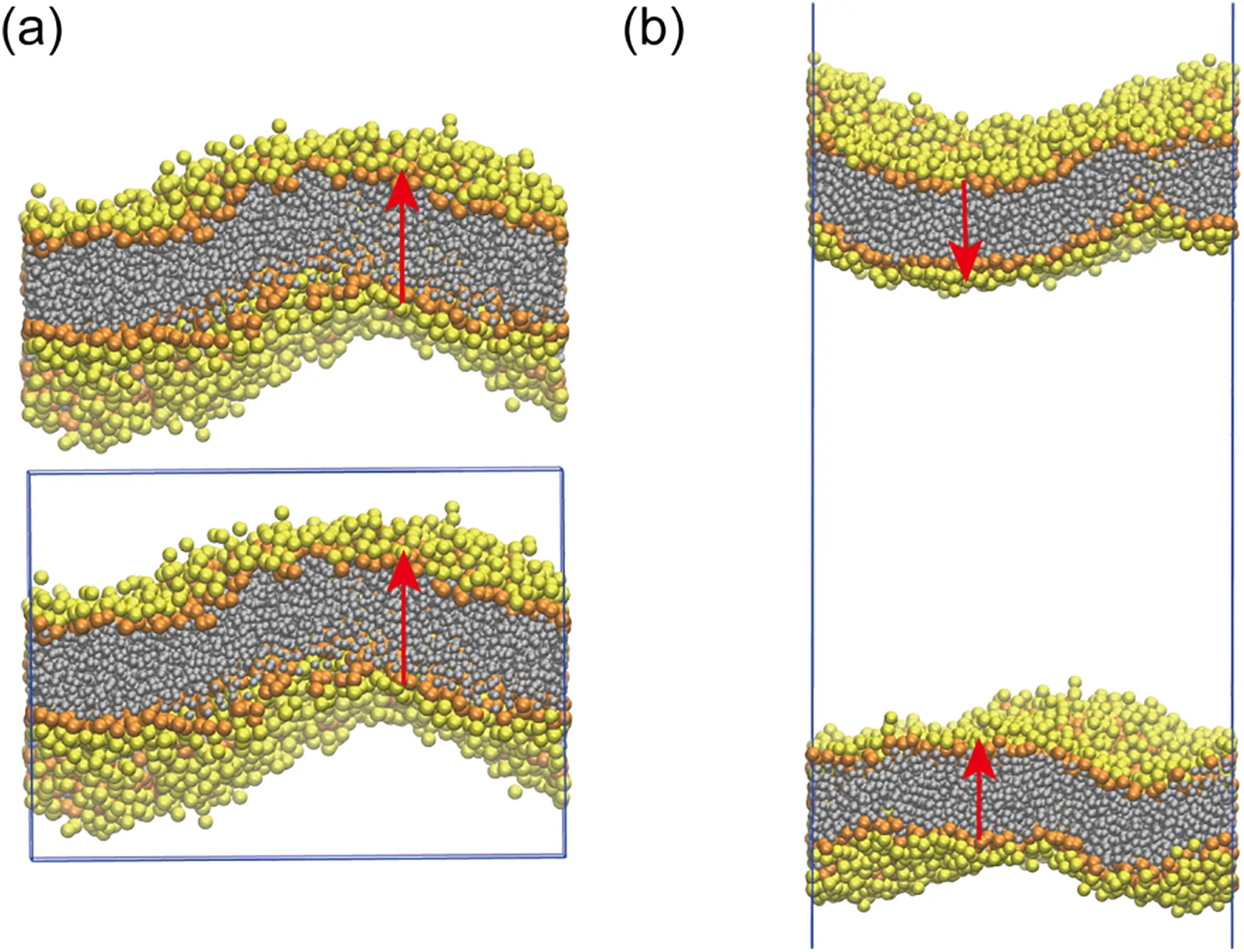
Comparison of deformation characteristics of single membrane and double membranes under the influence of the electric field. (a) Deformation fluctuation of single membrane under periodic boundary conditions; (b) Deformation fluctuation of double membrane.

To further quantify the orientational characteristics of the single and double membranes under different electric field strengths, we employed a spatially resolved angular analysis method [62]. Specifically, the simulation box was divided into 1 nm × 1 nm subregions along the x and y directions, and in each subregion the angle between the phospholipid membrane normal and electric field direction was calculated. The fraction of subregions with angles below 45° was then determined. As shown in Fig 8, the corresponding fractions for the single membrane vs the double membrane were 58.7% vs 66.2% (0.2 V/nm), 62.9% vs 70.4% (0.4 V/nm), and 72.7% vs 76.4% (0.6 V/nm), with significant differences between the two groups (P < 0.0001).

**Fig 8:**
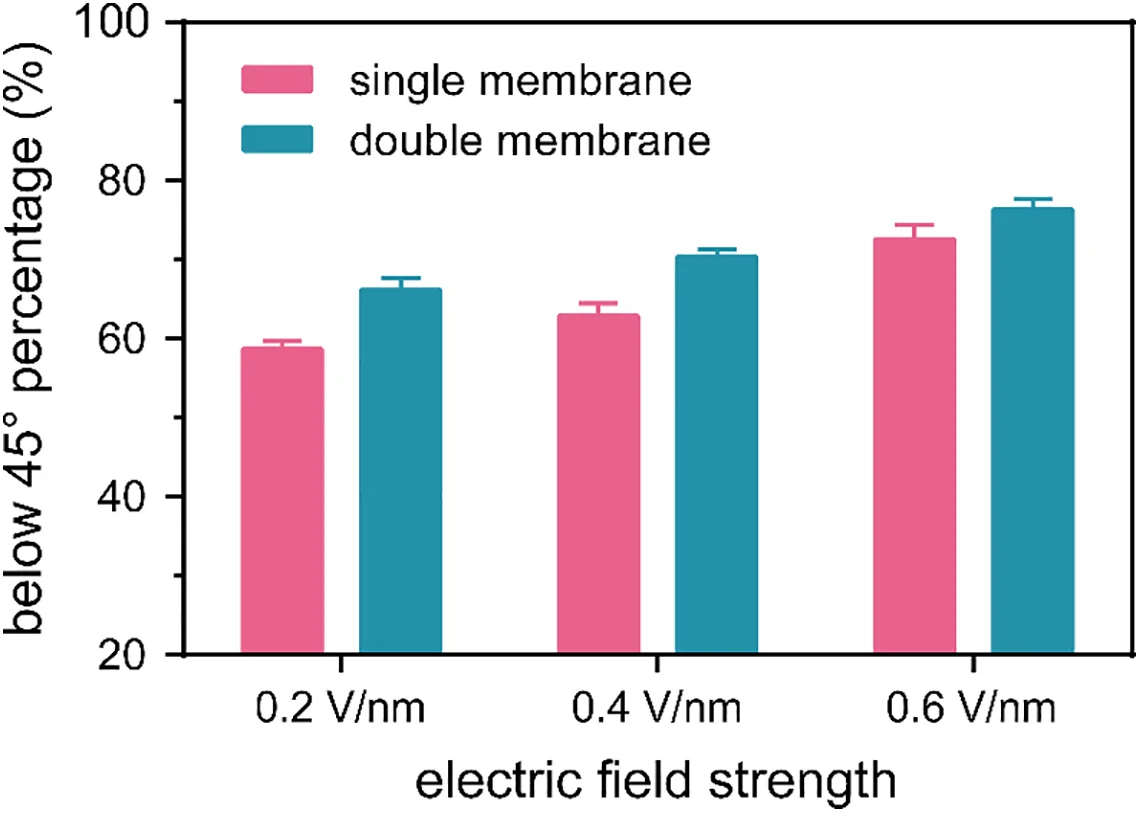
Analysis of phospholipid membrane orientation. The simulation box was divided into 1 nm × 1 nm subregions along the x and y directions. The angle between the phospholipid membrane normal and the z-axis was calculated in each subregion, and the percentage of subregions with angles below 45° was determined relative to the total number of subregions.

### 3.3 Facilitated pore formation driven by counter-deformation induced membrane tension loss

When the electric field angles tend toward smaller values, membrane tension is significantly reduced, thereby accelerating pore formation [60]. To further investigate whether the enhanced electroporation efficiency in the double membrane is driven by the electric field angle redistribution induced by intermembrane counter-deformation, we conducted the comparative analysis of membrane tension between the single and double membranes. The results showed that, with increasing electric field strength, membrane tension decreased in both systems, but the reduction was markedly greater in the double membrane (Fig 9). At the electric field strength of 0.2 V/nm, the membrane tension decreased by 10.87 mN/m with replicate values of −10.72 mN/m and −9.90 mN/m in the single membrane (S5 Fig in S1 File). The membrane tension decreased by 106.52 mN/m with replicate values of −102.92 mN/m and −90.96 mN/m in the double membrane. Statistical analysis indicated no significant differences among the replicates for either the single membrane or the double membrane. When the electric field strength increased to 0.6 V/nm, the tension dropped by 277.36 mN/m in the single membrane and by 741.01 mN/m in the double membrane. These findings indicate that compared to the single membrane, the unique counter-deformation of the double membrane, which promotes interleaflet convergence, can significantly reduce membrane tension. Notably, positive and negative surface tension values denote the relative change in membrane surface tension with respect to the zero-field state. Membrane electroporation is governed by the combined effects of membrane tension, electric field strength, and exposure time; higher membrane tension requires both higher electric field strength and longer exposure time to induce electroporation. These findings indicate that, compared with the single membrane, the counter-deformation specific to the double membrane, which promoted interleaflet convergence, shifted the membrane normal toward smaller angles relative to the electric field. This reduced the membrane tension governed by Coulomb interactions, lowered the pore formation energy barrier, and ultimately accelerated electroporation. Electroporation, as a non-thermal ablation technique, was analyzed without considering temperature effects in this study. However, research has shown that increased electrical conductivity due to temperature elevation can enhance the electroporation effect [63]. Moreover, since rising temperature also reduces membrane tension, it may further promote electroporation through both increased conductivity and decreased membrane tension.

**Fig 9:**
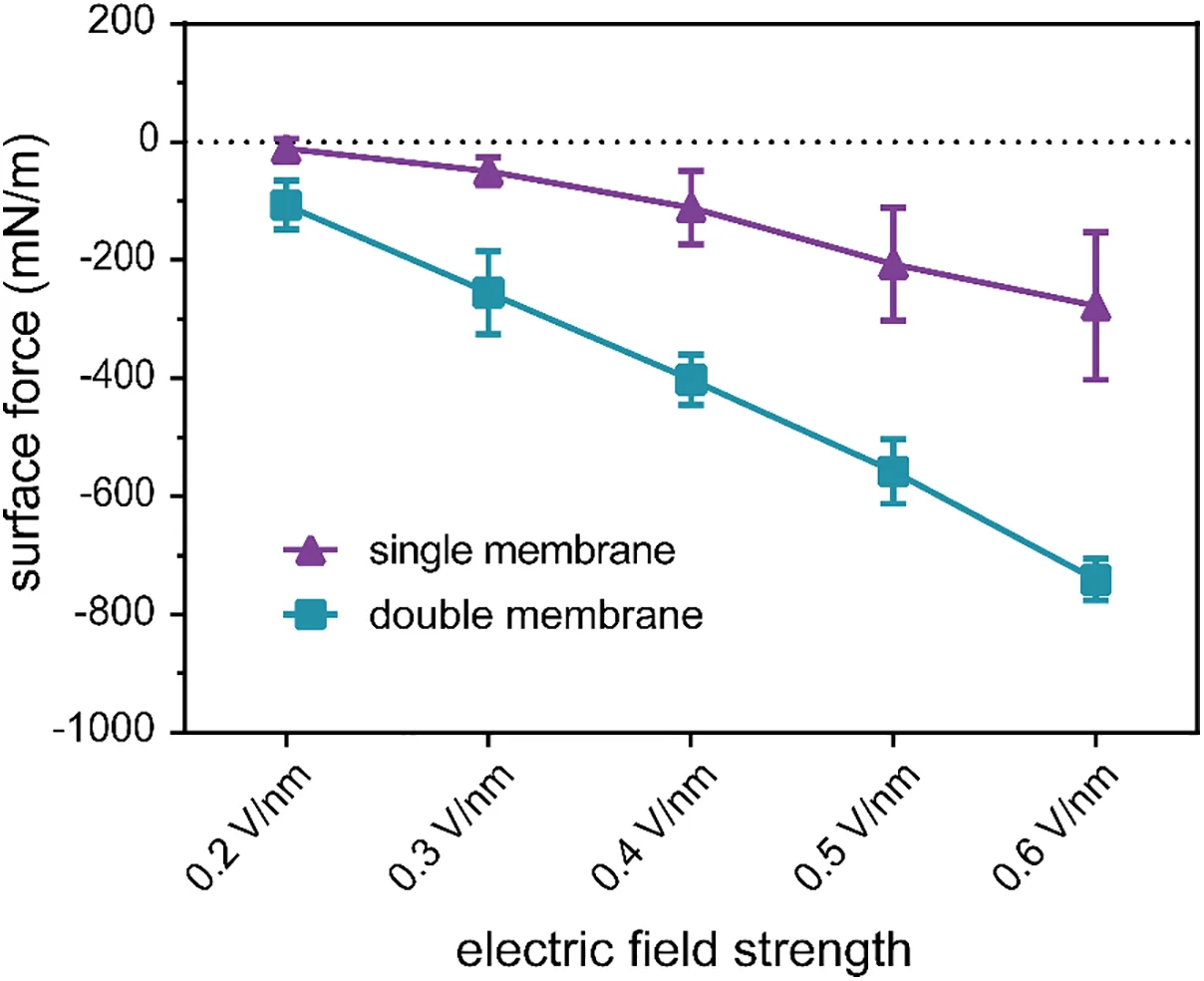
Comparison of membrane tension between the single and double membranes under different electric field strengths. The purple and blue lines represent the single membrane and double membrane, respectively. Error bars represent standard deviation.

To further elucidate the causal relationship between membrane tension reduction and pore formation, the evolution of the double membrane tension over time under the lowest (0.2 V/nm) and highest (0.6 V/nm) electric field strengths was analyzed (Fig 10). At 0.2 V/nm, the double membrane tension continuously decreased during the pre-pore stage (0–0.6 ns) and reached the critical threshold at 0.6 ns, coinciding with the time of pore formation. A similar trend was observed at 0.6 V/nm: membrane tension decreased over time, reaching a threshold at 0.2 ns that induced pore formation. Once the pore expanded beyond the scale of the simulated system (after ~0.5 ns), the membrane tension stabilized. These findings further confirm that reduced membrane tension promotes pore formation. In addition, electric-field-induced membrane remodeling may involve local field redistribution and preferential pore formation [64]. Thus, membrane tension alone may underestimate intermediate-state contributions. Future work should use enhanced sampling to identify critical remodeling conditions.

**Fig 10:**
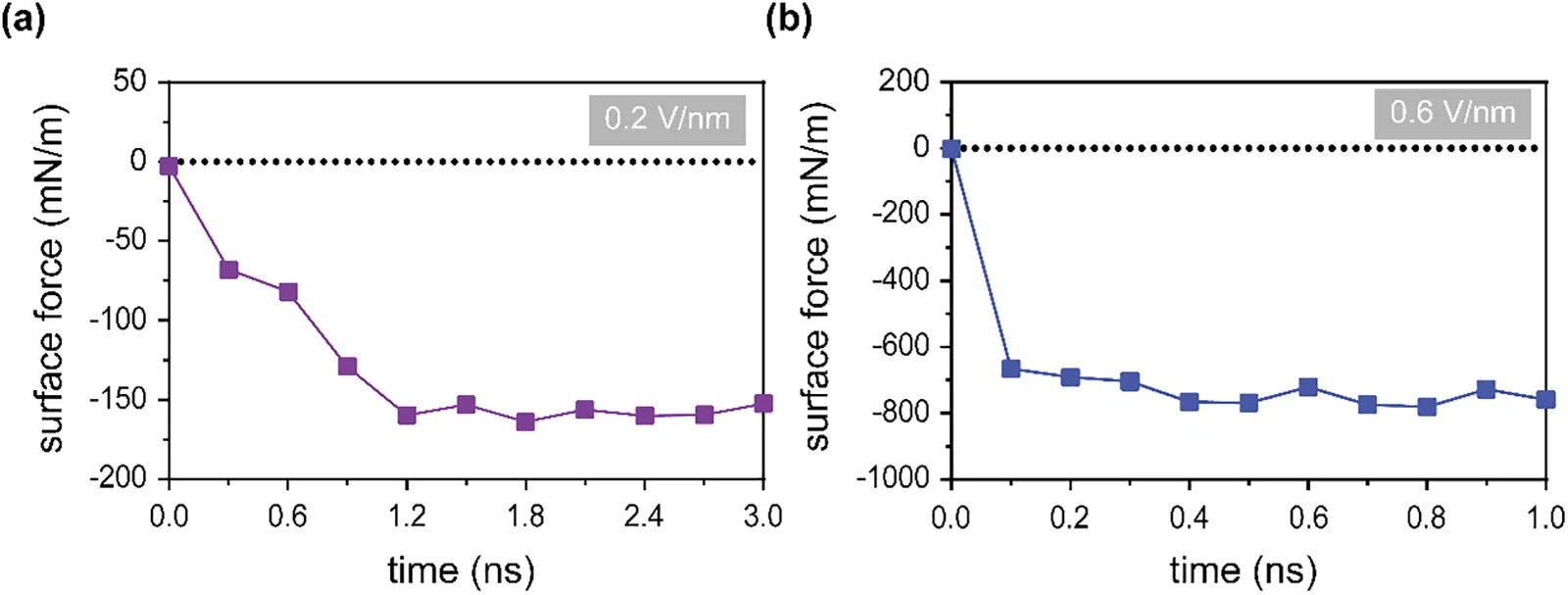
Change in membrane tension of the double membrane with different electric field durations. (a) 0.2 V/nm. (b) 0.6 V/nm. The time was measured from the application of the electric field.

## 4. Conclusions

Electroporation with nsPEFs has garnered increasing attention for its potential in tumor ablation. The electroporation effects on both the plasma membrane and the nuclear envelope play crucial roles in inducing irreversible cell damage. To elucidate the different effects of nsPEFs on these two membranes at the microscopic level, this study employed MD simulations to compare the electroporation responses of the plasma membrane model (single membrane) and the nuclear envelope model (double membrane) under the same electric field strengths. The results showed that under the same electric field conditions, the pore formation time for the double membrane was significantly shorter than that for the single membrane. Further analysis revealed that this outcome stemmed from the unique counter-deformation between the two bilayers of the double membrane, which altered the local electric field angle distribution, thereby reducing membrane tension and significantly enhancing the electroporation effect. This finding reveals that nsPEFs have a greater damaging effect on the double membrane of the nuclear envelope at the microscopic mechanistic level. In this study, both the plasma membrane and nuclear envelope were represented using a coarse-grained pure DPPC model, thereby neglecting key features that are highly relevant to electroporation behavior, such as membrane compositional asymmetry, nuclear envelope proteins, and the mechanical coupling associated with the nuclear lamina. Consequently, direct extrapolation from this model to the native nuclear envelope is inherently limited. Future studies should progressively incorporate asymmetry, cholesterol, and protein components to establish more biologically realistic models of membrane electroporation.

## Supporting information

S1 File Model validation and additional analyses; Coulomb forces along XYZ directions for the single and double membranes at 0.2 V/nm from three replicates; Membrane tension for the single and double membranes at 0.2 V/nm from three replicates. (DOCX)
