# Supplementary material for: Tension reduction from inter-membrane counter-deformation accelerates nuclear electroporation
Source: PLoS One. 2026 Oct 23;21(10):e0361048. doi: 10.1371/journal.pone.0361048 (PMC13693903; doi:10.1371/journal.pone.0361048)
Supplement: Supplementary file 1 [file pone.0361048.s001.docx]

**Supporting Information: Tension reduction from** **inter-membrane** **counter-deformation accelerates nuclear electroporation**

Yuepeng Diao^1^, Ping Ye^1^, Baolin Liu^1,*^

1	School of Health Science and Engineering, University of Shanghai for Science and Technology, Shanghai, China.

*	Corresponding author

E-mail: blliuk@163.com

Contens

[1. Model Validation and Additional Analyses	1](#_Toc843)

[1.1 Simulation model configurations	1](#_Toc6564)

[1.2 Effect of Water-Layer Thickness on Electroporation Behavior	2](#_Toc26549)

[1.3 Effect of the Coarse-Grained Force Field on Electroporation Behavior	3](#_Toc29600)

[2. Coulomb forces along XYZ directions for the single and double membranes at 0.2 V/nm from three replicates	4](#_Toc12599)

[3. Membrane tension for the single and double membranes at 0.2 V/nm from three replicates	4](#_Toc18777)

[References	5](#_Toc28529)

2. **Model Validation and Additional Analyses**

**1.1 Simulation model configurations**

To verify the reliability of the comparison between the single and double membrane systems, a thickened-water membrane system and an all-atom membrane system were further constructed using the single membrane system as the reference. Under the same electric field conditions, their pore formation times were compared with those of the reference system to assess the potential effects of water-layer thickness and force-field selection on electroporation behavior. The detailed model parameters of the single membrane system, thickened-water membrane system, and all-atom membrane system are summarized in S1 Table. The molecular configuration of the all-atom membrane system is shown in S1a Fig, and the mass density profiles of the system components after equilibration are presented in the S1b Fig. The observed distribution characteristics are consistent with previous reports [1, 2].

TABLE S1. Details of the simulation box used for MD simulation.

|  | Single membrane system | Thickened-water membrane system^a^ | All-atom membrane system^b^ |
| --- | --- | --- | --- |
| DPPC | 1024 | 1024 | 1024 |
| NaCl | 0.15 M | 0.15 M | 0.15 M |
| Size | 17.3× 17.3 × 12.5 nm^3^ | 17.3× 17.3 × 35.9 nm^3^ | 17.1 × 17.1 × 8.5 nm^3^ |
| force field | Martini | Martini | Charmm36 |

a: The thickened-water membrane system, derived from the single membrane system by adding top/bottom 16.4nm water layers;

b: The all-atom membrane system, derived from the single membrane system via the all-atom force field.

**FIG S1.** **All-atom membrane system structure and density profiles of each component.** (a) All-atom membrane system. Blue and red beads represent the lipid membrane, while green, gray, and white beads denote water molecules, Na⁺ ions, and Cl⁻ ions, respectively. (b) Mass density profiles of each component in the all-atom membranesystem. Blue, red, green, and brown curves represent DPPC head groups, DPPC tails, water molecules, and ions, respectively.

**1.2 Effect of Water-Layer Thickness on Electroporation Behavior**

To exclude the potential effect of differences in water-layer thickness under periodic boundary conditions (PBC) on the comparison of electroporation behavior between the single membrane and double membrane systems, the thickened-water membrane system was constructed based on the single membrane system. Water layers with a thickness of 16.4 nm were added to both sides of the single membrane system, resulting in a total water-layer thickness under PBC that exceeded 50% of the intermembrane water-layer thickness in the double membrane system. All other simulation parameters were kept identical to those of the single membrane system.

Three independent simulations of the thickened-water membrane system were performed at electric field strengths of 0.3 V/nm and 0.6 V/nm, respectively. As shown in S2 Fig, at 0.3 V/nm, the pore formation time of the thickened-water membrane system was 0.77 ± 0.05 ns, which did not differ significantly from that of the single membrane system (0.90 ± 0.00 ns; P > 0.05). At 0.6 V/nm, the pore formation time was 0.23 ± 0.05 ns, which likewise did not differ significantly from that of the single membrane system (0.30 ± 0.00 ns; P > 0.05). These results indicate that variations in water-layer thickness do not significantly affect the comparison of pore formation times between the single and double membrane systems.

**FIG S2.** Comparison of the pore formation time between the single membrane system and the thickened-water membrane system under different electric field strengths. The red and blue bars represent the single membrane and the thickened-water membrane, respectively. Error bars represent standard deviation.

**1.3 Effect of the Coarse-Grained Force Field on Electroporation Behavior**

Considering that electrostatic interactions in the coarse-grained model may be softened at the highest electric field strength, an all-atom membrane system was constructed using the CHARMM36 force field [3], with temperature and pressure conditions identical to those used in the coarse-grained simulations.

Three independent simulations of the all-atom membrane system were performed at electric field strengths of 0.3 V/nm and 0.6 V/nm, respectively. As shown in S3 Fig , at 0.3 V/nm, the pore formation time was 1.14 ± 0.04 ns, which did not differ significantly from that of the single membrane system (0.90 ± 0.00 ns; P > 0.05). At 0.6 V/nm, the pore formation time was 0.32 ± 0.02 ns, also showing no significant difference from that of the single membrane system (0.30 ± 0.00 ns; P > 0.05). These results indicate that the softened electrostatic interactions in the coarse-grained force field do not significantly affect pore formation times.

It should be noted that this study did not monitor solvent responses under the highest electric field (e.g., excessive water alignment, directional water drift, or bulk flow across the simulation box). Therefore, nonphysical artifacts cannot be ruled out as a potential influence on the apparent pore formation kinetics. Future work will further examine field-induced solvent effects and evaluate their impact on the observed pore formation dynamics.

**FIG S3.** Comparison of the pore formation time between the single membrane system and all-atom membrane system under different electric field strengths. The red and green bars represent the single membrane and all-atom membrane, respectively. Error bars represent standard deviation.

3. **Coulomb forces along XYZ directions for the single and double membranes at 0.2 V/nm from three replicates**

**FIG S4.** **Coulomb forces along XYZ directions for the single and double membranes at 0.2 V/nm from three replicates.** The purple and blue bars represent the single membrane and double membrane, respectively. Error bars represent standard deviation.

4. **Membrane tension for the single and double membranes at 0.2 V/nm from three replicates**

**FIG S5. Membrane tension for the single and double membranes at 0.2 V/nm from three replicates.** The purple and blue lines represent the single membrane and double membrane, respectively. Error bars represent standard deviation.

**References**

[1] E.E. Ong, J.-L. Liow, The temperature-dependent structure, hydrogen bonding and other related dynamic properties of the standard TIP3P and CHARMM-modified TIP3P water models, Fluid Phase Equilibria 481 (2019) 55-65.

[2] R.M. de Souza, R.H. Ratochinski, M. Karttunen, L.G. Dias, Self-Assembly of Phosphocholine Derivatives Using the ELBA Coarse-Grained Model: Micelles, Bicelles, and Reverse Micelles, J Chem Inf Model 60(2) (2020) 522-536.

[3] J.B. Klauda, R.M. Venable, J.A. Freites, J.W. O'Connor, D.J. Tobias, C. Mondragon-Ramirez, et al., Update of the CHARMM all-atom additive force field for lipids: validation on six lipid types, J Phys Chem B 114(23) (2010) 7830-43.
